# A new malaria vaccination tool based on replication-competent *Plasmodium falciparum* parasites

**DOI:** 10.1038/s44321-024-00056-8

**Published:** 2024-03-21

**Authors:** Diana Moita, Miguel Prudêncio

**Affiliations:** Instituto de Medicina Molecular João Lobo Antunes, Faculdade de Medicina da Universidade de Lisboa, Lisboa, Portugal

**Keywords:** Microbiology, Virology & Host Pathogen Interaction

## Abstract

D. Moita and M. Prudêncio discuss the study by Goswami et al, in this issue of *EMBO Mol. Med*., on the generation and pre-clinical characterization of late liver stage-arresting genetically attenuated *Plasmodium* parasites as a potential vaccine against malaria.

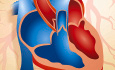

## Teaser

Recently licensed subunit vaccines represent the first and, thus far, the only approved agents for vaccination against malaria. However, these vaccines still fail to confer highly effective long-lasting protective immunity. Whole-organism vaccines, employing attenuated *Plasmodium* sporozoites as immunization agents, constitute a promising alternative for highly effective malaria vaccination. In this issue of *EMBO Molecular Medicine*, Goswami et al (2024) report on the generation and pre-clinical characterization of genetically attenuated *Plasmodium* parasites, termed LARC2, whose development arrests at late stages of liver infection. Their results warrant the clinical evaluation of PfSPZ-LARC2 towards its use as a whole-organism vaccine against malaria.

While malaria continues to affect millions of individuals and claim thousands of lives every year (WHO, 2023), the World Health Organization (WHO) has defined a strategic plan aimed at reducing the global incidence and mortality rates associated with this parasitic disease by at least 90% by 2030 (WHO, 2021). The recently licensed RTS,S/AS01 and R21/Matrix-M subunit vaccines targeting the pre-erythrocytic stage of *Plasmodium falciparum* (*Pf*) represent the first and, thus far, the only successful vaccines to target a human parasite (Oduoye et al, 2024). However, although the latter demonstrates seemingly enhanced protective efficacy compared to the former, both vaccines still fall short of meeting the goals set by the WHO, particularly in terms of long-term protection (Oduoye et al, 2024; WHO, 2021). Moreover, these vaccines target a single parasite species and a single parasitic antigen, the *Pf* circumsporozoite protein, whose allele-specific variations may result in the emergence of parasites that evade the vaccine-induced immunity. Also, since both RTS,S/AS01 and R21/Matrix-M only counteract clinical manifestations of the disease, they do not prevent further transmission of the parasite to the mosquito vector (Oduoye et al, 2024). Therefore, current research is focused on developing second-generation vaccines with improved characteristics and greatest impact, capable of blocking *Plasmodium* infection, reducing transmission, and protecting against multiple species, particularly *Pf* and *P. vivax*.

Vaccination with whole *Plasmodium* sporozoites potentially represents a significant stride relative to currently available vaccines. Whole-sporozoite (WSpz) immunization strategies target a broader range of antigens than subunit vaccines and have consistently been shown to not only prevent malaria-associated clinical symptoms but also block infection and, thus, cease further transmission (Nunes-Cabaco et al, 2022). The clinically most advanced WSpz vaccine is the Sanaria® PfSPZ Vaccine, composed of aseptically purified, cryopreserved radiation-attenuated *Pf* sporozoites (RAS). Importantly, a wide range of human clinical trials have demonstrated that this vaccine affords protection against homologous and heterologous controlled human malaria infection (CHMI) in malaria-naive individuals in the USA and Europe, as well as against natural infection in malaria-exposed adults in Africa (Nunes-Cabaco et al, 2022). In addition, vaccination employing non-attenuated *Pf* sporozoites under chemoprophylaxis with an antimalarial drug, such as chloroquine (PfSPZ-CVac), afforded 100% sterile protection against heterologous CHMI with a considerably lower dose than the PfSPZ Vaccine (Nunes-Cabaco et al, 2022). This could be attributed to an increase in both the quantity and diversity of parasitic antigens expressed throughout the entirety of the liver stage which is severely limited in the PfSPZ Vaccine sporozoites due to radiation-induced DNA damage (Nunes-Cabaco et al, 2022). Genetically-attenuated parasites (GAPs), in which the expression of one or more genes crucial for the completion of the parasite’s liver stage development has been abolished, have emerged and more recently started to undergo clinical assessment (Nunes-Cabaco et al, 2022). As a result of the targeted deletion of these genes, the parasites’ hepatic development is arrested at defined points, without establishing a symptomatic blood-stage infection. Similarly to RAS, early-arresting replication-deficient (EARD) GAPs are able to invade hepatocytes but abort development shortly afterward (Nunes-Cabaco et al, 2022). The PfSPZ-GA1, generated by deleting the *Pf b9* and *slarp* genes, was the first *Pf* EARD GAP to undergo clinical evaluation, achieving sterile protection levels that were utterly discouraging (Nunes-Cabaco et al, 2022).

As an alternative, *Pf* GAPs that retain their replication competence and exhibit a delayed growth arrest in the liver are expected to outperform the aforementioned strategies owing to a multitude of factors (Nunes-Cabaco et al, 2022). While the PfSPZ-CVac is inherently a more potent immunogen than the PfSPZ Vaccine, the report of side effects associated with transient parasitemia after the first dose (Nunes-Cabaco et al, 2022), and intrinsic safety issues related to the administration of fully infectious *Pf* sporozoites that require chemo-attenuation represent potential setbacks for the field implementation of this vaccination strategy. Hence, late-arresting replication-competent (LARC) parasites combine the intrinsic and irreversible attenuation phenotype of EARDs with the increased immunogenic potential of the PfSPZ-CVac, as the immunizing agent’s liver stage development halts at a late stage, exposing the host’s immune system to a broader antigenic repertoire than either RAS or EARDs. Moreover, GAPs can undergo additional genetic modifications, enabling the expression of transgenes that increase the vaccine’s immunogenicity and/or target different *Plasmodium* species. Very recently, a direct comparison performed by our team on the short- and long-term protective efficacies elicited by four distinct rodent malaria WSpz vaccines revealed that EARD provides the lowest overall protection throughout the 8-month period of assessment, while RAS and LARC induced the most durable protection, even when compared with chemo-attenuated sporozoites (Moita, Nunes-Cabaco, et al, 2023; Moita, Rola et al, 2023). In fact, the improved protective efficacy of LARC relative to EARD has already been confirmed in the clinic, with the demonstration that *Pf mei2-* (PfSPZ-GA2) induced significantly higher protective immune responses in humans than PfSPZ-GA1 (Franke-Fayard, 2022).

In this issue of *EMBO Molecular Medicine*, Goswami et al (2024) report on the generation and comprehensive characterization of a new vaccine candidate against malaria (Goswami et al, 2024). Through functional expression screening of late liver-stage parasites, they identified two *Plasmodium* genes, *mei2* and *linup*, essential for late-stage parasite development in the liver. Simultaneous deletion of these genes in the rodent-infective *P. yoelii* (*Py*) using CRISPR/Cas9-mediated gene editing resulted in a parasite, termed PyLARC2, which exhibits a complete defect during pre-erythrocytic development, preventing initiation of blood-stage infection. Importantly, immunization of rodents with PyLARC2 through different immunization routes induced sterile protection against the pre-erythrocytic stage of infection. Of particular relevance, intramuscular administration of PyLARC2 revealed high levels of protective efficacy. This may appear as somewhat surprising since a previous study by Epstein et al (2011) demonstrated that intramuscular administration of the PfSPZ Vaccine was significantly less effective than intravenous inoculation, likely because a higher number of sporozoites effectively reach the liver in the latter case, enhancing the hepatic antigen load that prime liver-resident immune responses (Epstein et al, 2011). However, the exact mediators and mechanisms underlying the protection conferred by the intramuscularly administered LARC2 remain to be explored, and whether this administration route also engenders long-lasting sterile protection, as observed with the intravenous route is yet to be ascertained. Nevertheless, if similar mechanisms are found to be present in humans, these findings might significantly impact the field of WSpz vaccines, as intravenous administration can be logistically complex and technically challenging.

This study also reveals that pre-erythrocytic immunization with PyLARC2 can elicit both cross-stage and cross-species protective immune responses, indicating the presence of antigens shared between liver and blood stages of infection and between distinct *Plasmodium* species, respectively (Goswami et al, 2024). Interestingly, however, the authors observed that late liver-stage PyLARC2 parasites lacked expression of MSP-1 despite this antigen being present not only in blood-stage parasites but also in sporozoite and liver stages (Nahren**d**orf et al, 2015). This suggests that other antigens shared between these stages may contribute to the observed stage-transcending protection, highlighting the potential utility of whole-organism immunizing agents such as LARC2 to uncover yet unidentified cross-stage protective antigens. Moreover, the authors found that both stage- and species-transcending protection could be in part antibody-mediated, although the potential involvement of cellular immune responses remains to evaluated (Goswami et al, 2024).

Following the completion of the PyLARC2 immunogenicity and protective efficacy study, the authors deleted the orthologous *mei2* and *linup* genes in the *Pf* genome to generate the PfLARC2 parasite (Goswami et al, 2024). Cryopreservation of PfLARC2 sporozoites employing Sanaria’s methods for PfSPZ research products yielded the PfSPZ-LARC2 vaccine candidate, which was found to exhibit a developmental arrest phenotype in a humanized mouse model similar to that displayed by its *Py* counterpart in wild-type rodents (Goswami et al, 2024).

In summary, the current study demonstrated that (i) PySPZ-LARC2 parasites display a hepatic arrest phenotype similar to that of the non-cryopreserved PyLARC2 line and, most importantly, that they induce sterilizing immunity against an infectious sporozoite challenge; and (ii) PfSPZ-LARC2 parasites, generated through the deletion of the *mei2* and *linup* genes in the *Pf* genome, display a liver development phenotype similar to that of its *Py* counterpart. Both these pre-clinical observations warrant the GMP-compliant manufacture and vialing of PfSPZ-LARC2 for clinical assessment and support its further development for use as a WSpz vaccine.
